# Orbital manifestations of inflammatory bowel disease – a systematic review

**DOI:** 10.1007/s00417-026-07309-x

**Published:** 2026-06-08

**Authors:** Charlotte Zhang, Terence Ang, Abdullah Almater, Dinesh Selva

**Affiliations:** 1 School of Medicine, University of Adelaide, Adelaide, SA 5000 Australia; 2 Department of Ophthalmology, Royal Adelaide Hospital, Adelaide, SA 5000 Australia; 3 Research Excellence Centre in Ophthalmology and Visual Sciences, Department of Ophthalmology, College of Medicine, King Saud University, Riyadh, Saudi Arabia; 4 King Saud University Medical City, King Saud University, Riyadh, Saudi Arabia; 5 South Australian Institute of Ophthalmology, University of Adelaide, Adelaide, SA 5000 Australia; 6 School of Medicine, Faculty of Health Sciences and Medicine, University of Adelaide, Adelaide, Australia

**Keywords:** Orbital inflammation, Inflammatory Bowel Disease, Extra-intestinal manifestations, Orbital inflammatory syndrome

## Abstract

**Background:**

Extra-intestinal manifestations of inflammatory bowel disease (IBD) occur in 25–40% of patients; however, orbital involvement is rare and underreported. Only case reports and series have been documented in the literature with no consensus on diagnosis or management.

**Aim:**

This systematic review aims to compile and analyse the published literature on orbital manifestations of IBD, focusing on clinical presentation, imaging, management and outcomes.

**Methods:**

A systematic search of PubMed, Embase, and Web of Science was performed from database inception to April 2025. Data were collected and risk of bias was assessed using the JBI Critical Appraisal Checklist for Case Reports/Case Series.

**Results:**

41 studies reporting 48 patients were included. Mean age at orbital presentation was 31 years (range 2–63 years) with a female predominance (3:1). Orbital inflammation preceded IBD diagnosis in 19 patients. Crohn’s disease was most frequently associated (*n* = 40). The most common clinical features were periorbital pain, swelling and ophthalmoplegia, with orbital myositis (*n* = 32) and dacryoadenitis (*n* = 15) being the predominant manifestations. 30 of 48 cases (63%) presented with bilateral disease, in contrast to the 8–20% bilateral rate typically reported for non-specific orbital inflammation (NSOI). Imaging most often demonstrated extraocular muscle enlargement. The majority of patients received systemic corticosteroids, often in combination with immunomodulators or biologics with most achieving remission. Recurrence occurred in 22 patients, and two experienced permanent vision loss.

**Conclusion:**

Orbital IBD is a rare but clinically significant entity, and may precede intestinal symptoms, sometimes representing the first sign of underlying IBD. Clinicians should maintain vigilance for orbital involvement in IBD, and standardised prospective reporting is needed to better characterise these manifestations and optimise patient outcomes.

**Supplementary Information:**

The online version contains supplementary material available at https://doi.org/10.1007/s00417-026-07309-x.

## Introduction

Inflammatory bowel disease (IBD), encompassing Crohn’s disease (CD) and ulcerative colitis (UC), is a common chronic inflammatory condition of the gastrointestinal tract. It demonstrates a bimodal age distribution, peaking at 15–30 years, and again at 50–80 years. CD is slightly more common in females, while UC shows a slight male predominance [1]. While IBD primarily affects the bowel, extra-intestinal manifestations (EIMs) occur in 25–40% of patients, involving the skin, joints, liver, kidneys, and eyes [2]. Ocular manifestations are the third most common EIM [3], affecting 4–12% of individuals, more frequently in CD than UC [4]. The most common are episcleritis, scleritis, and anterior uveitis [5]. However, orbital involvement is exceptionally rare and underreported [6, 7]. Orbital manifestations include orbital myositis [8], dacryoadenitis [9, 10] and orbital masses [11], and less commonly, dacryocystitis, superior ophthalmic vein thrombosis (SOVT), and nasolacrimal duct obstruction (NLDO) [12]. The pathophysiology of IBD-associated orbital inflammation remains unclear, and diagnosis is often delayed due to non-specific symptoms, which may mimic conditions such as infection or malignancy [13]. Additionally, orbital inflammation may precede IBD diagnosis.

Current knowledge of IBD-associated orbital inflammation is largely limited to case reports and small case series, with no consensus on diagnosis or management. Given the potential for diagnostic delays and the risk of irreversible visual loss, a synthesis of published cases is warranted. This systematic review summarises the literature on IBD-associated orbital inflammation, with a focus on clinical presentation, radiological characteristics, management, and outcomes.

## Methods

This systematic review was conducted in accordance with the Preferred Reporting Items for Systematic Reviews and Meta-Analyses (PRISMA) 2020 guidelines (Appendix 1).

### Search strategy

A systematic search of PubMed, Embase, and Web of Science was conducted from database inception to April 2025 to identify literature describing orbital manifestations in patients with IBD. Search terms were identified through preliminary scoping searches and included “inflammatory bowel disease”, “Crohn’s disease”, “ulcerative colitis”, “orbit”, “orbital”, “orbital manifestations”, “orbital myositis”, “dacryoadenitis”, “orbital inflammation”, and “orbital mass”, using Boolean operators to refine searches as appropriate (full strategy in Appendix 2).

Title and abstract screening were conducted independently by two reviewers (C. Zhang and T. Ang), with discrepancies resolved through discussion and consensus. Eligible studies subsequently underwent independent full text review by the same two reviewers, with disagreements regarding study inclusion resolved again through discussion and consensus. Studies were included if they involved patients with a confirmed diagnosis of IBD, reported orbital involvement (e.g. orbital myositis, lacrimal gland inflammation, orbital masses) and provided sufficient clinical, radiological or therapeutic detail for data extraction. Only English articles with full-text availability were considered. Exclusion criteria comprised: absence of IBD, lack of orbital pathology, isolated anterior segment ocular manifestations (i.e. episcleritis, uveitis, scleritis), insufficient extractable clinical data, editorials, comment, or conference abstract publications, review articles, and where full text was not available.

Included articles were analysed for patient demographics, IBD characteristics, clinical and radiological features, treatment modalities, and patient outcomes. Potential duplicate patient reports were assessed through comparison of author groups, institutions, patient demographics, clinical characteristics, and publication timelines. No definite duplicate cases were identified among the included studies. Risk of bias was assessed using the JBI Critical Appraisal Checklist for Case Reports/Case Series (study-level assessments provided in supplementary materials). A full list of extracted variables and individual case data is provided as supplementary data. Missing or unclear data were recorded as ‘not specified’.

Data from included studies were tabulated by patient demographics, IBD characteristics, treatment modalities and outcomes. Narrative synthesis was used to summarize patterns across cases, given heterogeneity of data and lack of comparable effect measures.

## Results

### Study characteristics

**Fig. 1:**
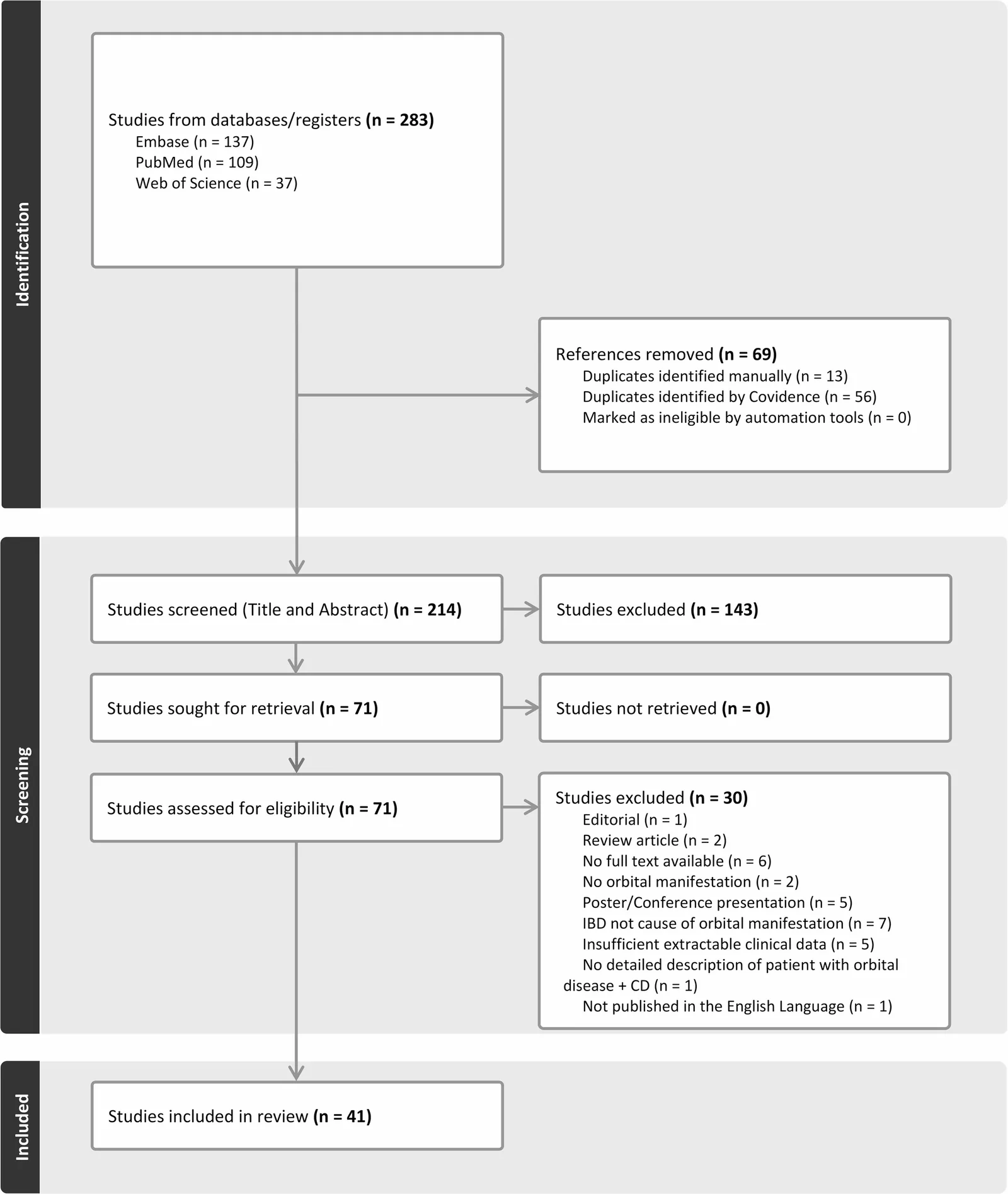
PRISMA flowchart illustrating flow of information and articles excluded during search

214 articles were screened, of which 41 met the inclusion criteria (Fig. 1) [7–47], comprising 48 patients (supplementary raw data) with orbital manifestations of IBD. Most cases originated from the USA (*n* = 20), the UK (*n* = 7), and Canada (*n* = 5), with other cases from Australia, Spain, Hungary, Turkey and France (*n* = 2 each), and Greece, Argentina, Croatia, Japan, Portugal and Singapore (*n* = 1 each). Publication year ranged from 1981 to 2025, with 16 cases reported in the past decade.

### Patient demographics

The mean age at orbital presentation was 31 years (range 2–63), with a female predominance (3:1) (Table 1). Orbital manifestations were more frequently associated with CD than UC (Table 2). The interval from IBD diagnosis to orbital presentation varied from 9 months to 29 years (mean 10.18 years; 3 cases unspecified). In 19 individuals, orbital inflammation preceded IBD diagnosis.

**Table 1:** Demographics of included patients (*n* = 48)

Variable	Result
Mean age at orbital presentation (years)	31 (range 2–63)
Median age at presentation (years)	32
Standard deviation	13.95
Sex (female: male)	36:12 (3:1)

**Table 2:** Disease characteristics and orbital findings in patients with IBD-related orbital manifestations (*n* = 48)

Category	Variable	Number of cases and percentage of total (%)
IBD type	Crohn’s Disease	40 (83)
	Ulcerative Colitis	7 (15)
	Non-specific focal active colitis	1 (2)
IBD activity at presentation	IBD diagnosis not yet established at time of orbital presentation	19 (40)
	Active disease	16 (33)
	Remission	11 (23)
	Unspecified	2 (4)
IBD therapy status	On maintenance therapy	28 (58)
EIMs	Musculoskeletal	10 (21)
	Dermatological	9 (19)
	Intraocular	7 (1 subclinical anterior uveitis + episcleritis, 1 posterior scleritis, 1 scleritis, 1 iridocyclitis, 2 episcleritis, 1 recurrent iritis) (15)

At presentation 16 patients had active disease at time of presentation, 11 were in remission, and 2 had unspecified disease activity. The majority (*n* = 28) were on maintenance therapy for IBD.

Extraintestinal involvement included musculoskeletal manifestations in 10 patients, dermatological in 9, and intraocular in 7. No hepatopancreatobiliary or renal involvement was reported.

Thyroid status was variably reported across included cases. Of 48 cases, 22 (45.8%) explicitly reported no evidence of thyroid disease or thyroid eye disease. One case reported coexisting thyroid disease. In the remaining 25 cases (52.1%), thyroid status was not specified.

### Clinical presentation

The most common presenting features were periorbital pain, periorbital oedema/swelling and ophthalmoplegia (Table 3). Of 31 cases reporting visual acuity, 19 had normal or unchanged vision, while the remainder had variable impairment (Table 4).

**Table 3:** Frequency of clinical features

Symptom	Number of cases and percentage of total (%)
Periorbital pain	35 (73)
Periorbital oedema/swelling	32 (67)
Ophthalmoplegia	26 (54)
Conjunctival injection	19 (40)
Diplopia	19 (40)
Proptosis	17 (35)
Painful ocular motility	16 (33)
Periorbital erythema	13 (27)
Chemosis	11 (23)
Blurred vision	11 (23)
Palpable orbital mass	8 (17)
Presence of optic neuropathy (e.g. relative afferent pupillary defect)	6 (13)
Visual Field loss	4 (8)

**Table 4:** Clinical characteristics of orbital presentation

Feature	Category	Number of cases (*n*)
Visual Acuity	Normal/unchanged	19
	6/6–6/9	4
	6/12 − 6/18	2
	6/24 − 6/60	2
	Counting fingers (CF) / Hand Movements (HM) / 6/120	3
	No light perception (NLP)	1 (unilateral in patient with superior ophthalmic vein thrombosis; Mandic, 2018).
Laterality of presentation	Unilateral	18
	Synchronous Bilateral	16
	Metachronous Bilateral	14
Recurrent episodes	Yes	22
Initial misdiagnosis	Yes	11 (7 bacterial infection, 4 other: Grave’s ophthalmopathy, idiopathic intracranial hypertension, hordeolum, scleritis.
Time from symptom onset to correct diagnosis		Ranging from a few days to 10 months in misdiagnosed cases.

Presentation was most commonly unilateral (*n* = 18), or synchronously bilateral (*n* = 16), with fewer metachronously bilateral cases (*n* = 14). Recurrent episodes occurred in 22 patients, and 11 were initially misdiagnosed, most frequently as bacterial infection.

### Orbital manifestations

Orbital myositis was the most frequently reported manifestation (*n* = 32), followed by dacryoadenitis (*n* = 15) (Table 5). Other findings included NLDO, dacryocystitis, optic perineuritis/neuritis, pyoderma gangrenosum, and SOVT. Orbital myositis/diffuse extraocular muscle (EOM) enlargement, or dacryoadenitis typically presented as a non-specific ‘orbital mass’.

**Table 5:** Reported orbital manifestations

Manifestation	Number of cases (*n*)
Orbital myositis	32
Dacryoadenitis	15
NLDO/Dacryocystitis	4 (3 NLDO, 1 dacryocystitis)
Optic perineuritis/neuritis	2
Pyoderma gangrenosum	1
Superior Ophthalmic Vein Thrombosis (SOVT)	1

### Radiological findings/histopathological identification

Imaging was performed in 47 patients (modality not specified in one case), most commonly MRI (*n* = 25) or CT (*n* = 20) (Table 6).

**Table 6:** Imaging characteristics of orbital manifestations in IBD (*n* = 47) (Note one patient did not have imaging modality specified)

Category	Variable	Number of cases (*n*)
Imaging modality	MRI	25
	CT	20
	Dacryoscintigraphy (for NLDO)	1
	MSCT angiography (for SOVT)	1
Orbital fat involvement	Involved	2
EOM involvement	Involved	32
Single vs. multiple EOM involvement	Single muscle involvement	13
	Multiple involvement	19
Which EOM(s) involved	Lateral rectus	19
	Medial rectus	16
	Inferior rectus	12
	Superior rectus	11
	Superior oblique	6
	Inferior oblique	4
	Diffuse (all EOM)	3
	Contrast enhancement	19
	Tendon involvement	4
Dacryoadenitis		15
Optic nerve involvement		5
Nerve sheath involvement		1
Orbital mass (non-specific)		8
Nasolacrimal involvement		4

EOM involvement was the most frequent imaging finding (*n* = 32), often affecting multiple EOMs, typically recti over obliques, often with contrast enhancement (*n* = 19). Tendon involvement was poorly reported (4 involved of 9 cases). Optic nerve involvement was uncommon, with unilateral involvement in 5 cases. There was one case of optic perineuritis [33]. An ‘orbital mass’ was described in 8 cases.

Nasolacrimal involvement occurred in 4 cases (3 NLDO, 1 complicating dacryocystitis). NLDO was attributed to chronic inflammatory infiltration and fibrosis of the lacrimal sac in one case (Kemeny-Beke et al. Case 1) [12] and granulomatous infiltration and fibrosis of the lacrimal drainage system in another [23]. In the third case the mechanism was unspecified (Kemeny-Beke et al. Case 2) [12].

Histopathological confirmation was performed in 33% (*n* = 16) of cases, most commonly from the lacrimal gland(s) (*n* = 8), followed by EOM (*n* = 5), lacrimal sac(s) (*n* = 4), and periorbital tissue (*n* = 1). Findings often demonstrated non-caseating granulomatous inflammation that was necrotising [10, 13, 30] or suppurative [19, 30], with occasional multinucleated giant cells [13, 23, 28, 45]. Medium-vessel vasculitis was reported in one case [13], while others demonstrated lymphoplasmacytic or T-cell predominant infiltrates with variable fibrosis [11, 28, 31, 38, 44], and one with elevated IgG4/IgG ratio [38].

### Treatment modalities and treatment outcomes

Most patients received medical management only (*n* = 41) (Table 7), while 5 in addition to medical therapy underwent procedural interventions (dacryocystectomy [19], retrobulbar triamcinolone injection and radiotherapy (Garrity et al. Case 2) [22], drainage of suppurative material for orbital myositis [30], and dacryocystorhinostomy (DCR) [12, 23]).

**Table 7:** Treatment modalities, response, and follow-up outcomes

Category	Variable	Number of cases
Medical management	Total	46 (42 with systemic steroids)
	Medical only	41
	Combination of medical and surgical/procedural	5
Response to medical therapy (*n* = 46)	Complete resolution	31
	Partial improvement	10
	Nil response	1
	Not specified	4
Surgical/Procedural management	Total	7
	Surgical only	2
	Combination of medical and surgical	5
Response to surgical therapy (*n* = 7)	Complete resolution	2
	Partial improvement	3
	Not specified	2
Recurrence		22
Follow-up outcomes	Steroid free remission	33
	Partial Improvement	6
	Permanent visual loss	2
	Remission with ongoing steroid requirement	1
	Ongoing multiple recurrences	1
	Not specified	5

Of the 46 patients managed medically, the majority (*n* = 42) received systemic corticosteroids, often combined with immunomodulators or biologics targeting underlying IBD (e.g. mycophenolate, adalimumab, infliximab, cyclophosphamide, 5-ASA, methotrexate, budesonide, 6-mercaptopurine, mesalamine, minocycline and azathioprine). Four patients did not receive steroids: one due to intolerance (Monaghan et al. Case 3) [13], one self-limiting [9], and two NLDO cases managed primarily with DCR with adjunct medical therapy [12, 23]. Most patients achieved steroid free remission, with immunomodulators or biologics were typically continued as maintenance therapy.

Best-corrected visual acuity (BCVA) was not consistently reported and therefore could not be used as a standardised outcome measure. Instead, narrative clinical descriptions provided in the included studies were used to classify treatment response. Of the 46 medically treated cases, 31 achieved complete resolution, 10 partial improvement (2 of these describing ‘significant improvement’;one case describing steroid therapy achieving ‘significant improvement in symptoms and orbital MRI findings’ [29], and the other describing a ‘significant improvement of bilateral orbital swelling’ [38]). One case showed no response to steroids [19]. The remaining 4 cases did not report response to medical therapy.

Surgical/procedural management was uncommon (*n* = 7), with 2 cases of sole surgical management (one resolving spontaneously after biopsy [28], and another case of NLDO treated solely with DCR [12]). NLDO was managed primarily with surgical intervention [12, 23].

Recurrence occurred in 22 cases, typically 1–3 recurrences, with 3 outliers experiencing more [38, 44, 47].

Follow-up ranged from one week to 13 years (though timing of reporting may account for this variability). Outcomes were unspecified in 5 cases. Of the remaining 43 cases, most achieved steroid free remission. 2 patients experienced permanent visual loss (pyoderma gangrenosum [19], SOVT [32]). One remained in remission on low dose steroids [36], and one had ongoing recurrences [47].

## Discussion

Orbital inflammatory disease (OID) encompasses inflammation of orbital structures [7], including the EOMs, lacrimal gland, orbital fat, or optic nerve/nerve sheath [48, 49]. OID is classified as non-specific orbital inflammation (NSOI), a diagnosis of exclusion [50], or specific, when secondary to systemic autoimmune/inflammatory disorders (e.g. IBD, IgG4-related disease, thyroid eye disease (TED) [7], sarcoidosis, granulomatosis with polyangiitis, SLE, Behcet’s disease) [51], infections (i.e. Lyme disease) [52] trauma, neoplasm, or drugs [53]. NLDO may arise secondary to orbital inflammation [54], while SOVT can develop in IBD [32] possibly due to systemic inflammation, hypercoagulability [55], or local factors [56].

This review identified 48 cases of IBD-associated orbital manifestations across 41 case reports/series. Orbital myositis was most common, followed by dacryoadenitis, with rarer reports of NLDO, dacryocystitis, pyoderma gangrenosum, and SOVT. Most patients were managed with systemic corticosteroids, with generally favourable outcomes. Recurrence was common but not associated with poorer outcomes.

The cases were mostly from the USA, UK and Canada, reflecting possible geographic reporting bias. The mean age of orbital presentation was 31, falling within the end of the first peak of IBD. The marked female predominance (3:1) may reflect the slight female predominance in CD [1], which comprised the majority of cases, or a true higher frequency of orbital manifestations in females. Orbital manifestations were overwhelmingly more common with CD than UC (40:7), likely reflecting the higher incidence of systemic EIM in CD [57, 58].

Orbital findings may often be the initial presenting symptom of underlying IBD. Ophthalmologists should screen for gastrointestinal symptoms in OID, particularly with recurrent episodes, and gastroenterologists should recognise orbital disease as a rare EIM. Unlike most EIMs, which generally correlate with intestinal IBD activity [59] (with exceptions [60, 61]), orbital inflammation showed no clear association, with near equal patients presenting during active disease and remission. Faecal calprotectin may have a role as a sensitive marker of subclinical intestinal inflammation in the absence of gastrointestinal symptoms [62] and could serve as a screening tool in patients presenting with OID. While corticosteroids are generally effective for orbital inflammation regardless of cause [63], recognition of IBD is important to guide long-term management.

Presenting features were generally non-specific and largely indistinguishable from NSOI [64], complicating diagnosis in some cases [22]. Laterality, however, was more balanced (bilateral involvement occurring in 63% of cases) unlike in NSOI, where unilateral presentations predominate (bilateral disease occurring in 8–20%) [64, 65]. Bilateral presentation may serve as a useful clinical clue, prompting consideration of IBD-associated orbital disease. Although visual outcomes are generally favourable; rare cases (SOVT, pyoderma gangrenosum) may progress to permanent visual loss [19, 32] (while almost all orbital myositis cases had favourable outcomes). Misdiagnosis as bacterial infection was common [14, 15, 21, 23, 30, 34, 42], delaying corticosteroid initiation and emphasising the importance of considering IBD in the differential for orbital inflammation, particularly in patients with systemic features.

The most common radiological finding was EOM enlargement, often affecting multiple recti, a pattern overlapping with TED [66]. While TED typically spares the tendinous insertions [18], the sparse reports of tendinous involvement limit meaningful comparison. Orbital fat and nerve involvement were rarely described, either reflecting true rarity or underreporting. Histopathological confirmation was infrequent, with biopsies of the lacrimal gland [10, 13, 28, 31, 38, 45], lacrimal sac [12, 19, 23], or EOMs [13, 30, 31, 44] demonstrating non-specific inflammatory infiltrates.

Systemic corticosteroids were the mainstay of treatment, generally producing favourable outcomes (complete resolution or partial improvement), though recurrence during tapering was frequent [7, 10, 19, 22, 31, 35, 38, 44, 47], necessitating escalation of steroid doses or the addition of steroid-sparing agents. Notably, prolonged use carried risks, including Cushingoid features and severe weight gain [11, 22], representing a therapeutic challenge of balancing treatment with adverse effects. Recurrent or refractory inflammation may prompt clinicians to search for a systemic cause even when the initial diagnosis is NSOI. Additional studies are required to determine the optimal steroid regimen, including the initial dose or course, and weaning rate. In addition, the efficacy of steroid-sparing agents [67] and adjunct treatments such as radiotherapy [68], that may reduce steroid-related adverse effects should be further investigated.

Recurrence frequency did not predict worse outcomes. Some patients with multiple recurrences ultimately achieved steroid-free remission [7, 10, 22, 35, 44, 69], while others with fewer episodes experienced irreversible vision loss [19, 32]. Recurrent episodes however, were associated with difficulty tapering steroids [47]. Overall, long-term outcomes were positive with most in steroid free remission, likely supported by adjacent use of immunomodulators and biologics. Infliximab (and other TNF-α inhibitors), commonly used for IBD and its EIMs, has been paradoxically associated with orbital inflammation [70, 71]. However, infliximab may be a useful option in refractory cases of orbital inflammation [72, 73]. A case series of seven patients with chronic treatment refractory orbital myositis (two with CD) reported improvement with infliximab [22]. A minority of cases did not require steroids or were self-limiting [9, 41] reflecting heterogeneity in disease severity. Surgery was rarely necessary, generally reserved for NLDO/drainage procedures/dacryocystitis [12, 23]. In isolated cases, bowel resection coincided with improvement or resolution of orbital inflammation, though causality is uncertain [22, 46].

This review has several limitations. All available evidence was derived from case reports and small case series, introducing publication bias with an overrepresentation of severe and unusual cases, limiting generalisability. There was significant heterogeneity of reporting, with frequent omission of key details such as tendon and orbital fat involvement, characterisation of optic nerve sheath involvement, and long-term outcomes. In particular, thyroid status was inconsistently reported across cases, limiting the ability to fully exclude concomitant thyroid eye disease as a confounding factor in all reported cases. The small sample size also restricts the ability to draw strong statistical conclusions. Future research should focus on prospective reporting of orbital EIMs in IBD, ideally via international multicentre registries, and standardised imaging reporting to facilitate robust comparisons with other orbital inflammatory conditions.

## Conclusion

Orbital manifestations of IBD are rare but clinically significant, often mimicking other ocular conditions and OID, leading to diagnostic delay. Orbital findings may precede intestinal symptoms, sometimes representing the first sign of underlying IBD. While most cases respond well to corticosteroids, recurrence is common and a rare minority progress to permanent vision loss. Recognising the systemic context is essential to avoid misclassification and guide long-term management. Greater awareness is required among ophthalmologists and gastroenterologists, and improved standardised prospective reporting is needed to better characterise these manifestations and optimise outcomes.

## Supplementary Information

Below is the link to the electronic supplementary material.

Supplementary Material 1 (DOCX 29.4 KB)

Supplementary Material 2 (DOCX 14.3 KB)

Supplementary Material 3 (XLSX 10.2 KB)

Supplementary Material 4 (XLSX 9.35 KB)

Supplementary Material 5 (XLSX 92.1 KB)

## Data Availability

Raw data is provided as supplementary material. No datasets were generated or analysed during the current study.
