# Supplementary material for: Orbital manifestations of inflammatory bowel disease – a systematic review
Source: Graefes Arch Clin Exp Ophthalmol. 2026 Jun 8;264(10):2979–88. doi: 10.1007/s00417-026-07309-x (PMC13635046; doi:10.1007/s00417-026-07309-x)
Supplement: Supplementary file 2 [file 417_2026_7309_MOESM2_ESM.docx]

**Appendix 2: Search strategy for systematic review**

Search Strategy

| IBD | Orbital inflammation |
| --- | --- |
| “Inflammatory bowel diseases” [mh] OR Crohns disease [tiab] OR Ulcerative colitis [tiab] | “Orbital diseases” [mh] OR “Orbital Myositis” [mh:noexp] OR “Dacryocystitis” [mh:noexp] OR ("inflammation"[mh:noexp] AND "orbit"[mh:noexp]) OR Orbit* inflammat* [tiab] OR orbit* myositis [tiab] OR dacryoadenitis [tiab] |
